# Empirical Solution of Stress Intensity Factors for the Inclined Inner Surface Crack of Pipe under External Pressure and Axial Compression

**DOI:** 10.3390/ma16010364

**Published:** 2022-12-30

**Authors:** Xi-Ming Yao, Yu-Chen Zhang, Qi Pei, Li-Zhu Jin, Tian-Hao Ma, Xiao-Hua He, Chang-Yu Zhou

**Affiliations:** 1 School of Mechanical and Power Engineering, Nanjing Tech University, Nanjing 211816, China; 2 Jiangsu Key Lab of Design and Manufacture of Extreme Pressure Equipment, Nanjing 211816, China

**Keywords:** inclined surface crack, stress intensity factor, pipe, crack closure, external and axial pressure, finite element analysis

## Abstract

Based on fracture mechanics theory, a finite element method was used to determine the stress intensity factors of the inclined crack on the inner surface of the pipe under axial compression load and external pressure. The effects of different influencing factors on the stress intensity factor along the crack front considering crack closure were systematically explored, which were different to those under internal pressure. The effects of high aspect ratio on *K_II_*, the crack inclination asymmetry caused by curvature and the effects of the friction coefficient on the stress intensity factors of the pipe with an inclined inner surface crack under axial compression load and external pressure were explored in this paper. To be fit for defect assessment, the solutions for stress intensity factors *K_II_* and *K_III_* were derived, and new correction factors *f_θ_* and *f_μ_* were proposed in the empirical solutions to accommodate the crack inclination asymmetry and the friction coefficient, respectively.

## 1. Introduction

Pipes are widely used in various fields of petroleum, chemical industry, and natural gas. In engineering practice, there are a large number of shells subjected to external pressure loads such as submarines, aerospace simulators, deep-sea pipelines, and buried pipelines. Due to the limitations of the manufacturing process, the structures may have tiny defects such as cracks. Lin and Smith [1] found that arbitrarily shaped cracks become semi-elliptical after a certain period of expansion. The stress intensity factor is an important fracture parameter for judging crack propagation and failure in defect assessment. Therefore, since the last century, scholars have conducted many studies on this subject.

In the early years, Raju and Newman [2] established a stress intensity factor empirical solution for mode I internal and external surface cracks of cylindrical vessels through FEA. The formula gave the influence coefficients based on different shell and crack sizes. Navid and Fenner [3] calculated the stress intensity factors of a thick-walled cylinder with internal pressure for different crack sizes based on the boundary integral equation method. Afterward, various numerical techniques were developed to calculate the stress intensity factor of pipe surface cracks such as the line spring model [4] and the weight function method [5,6,7]. Kamaya [8] combined the finite element alternating method and the finite element analysis to calculate the stress intensity factors of shell surface cracks. Wallbrink [9] proposed a semi-analytical method of conformal transformation to predict the stress intensity factors of circumferential surface cracks. However, most of these studies were limited to simple loading and the crack direction was axial or circumferential. In fact, in most cases, the loading conditions of the pipelines are complex and the crack direction is inclined. 

The research on mixed mode cracks first started from a flat plate. Murakami [10] analyzed *K_Ⅰ_*, *K_Ⅱ_*, *K_Ⅲ_* by the body force method, and provided a formula for the maximum value *K_θmax_* at the crack front according to the projected area of the crack in the direction of the maximum principal stress. Zeng and Dai [11] proposed a simplified analytical model of an inclined surface crack under biaxial stress, and proposed a closed solution for *K_Ⅰ_* and *K_Ⅲ_* at the deepest point of the crack front. Some scholars have also used FEA [12,13], the numerical influence function method [14], and the experimental method [15] to study the mixed mode cracks of flat plates. The study of mixed mode cracks in shell can be divided into two types. First, the crack direction is determined, usually axial or circumferential, and the mixed mode crack is formed by applying a complex far-field load on the structure. Shahani and Habibi [16] considered the stress intensity factor of cylinders with a circumferential crack under the action of axial force, bending moment, and torque. Predan et al. [17] studied the stress intensity factor for circumferential semi-elliptical surface cracks in a hollow cylinder subjected to pure torsion. Ramezani et al. [18] demonstrated the empirical solution of the stress intensity factor of cylinder surface crack under pure torsion. Second, the mixed mode cracks were formed by changing the inclination angle of the surface cracks. For example, Li et al. [19,20] studied the stress intensity factor of the inclined crack in the pipeline under the far field tension and tension-bending, and provided an empirical formula to calculate the complete value of the stress intensity factor at the crack front through the influence coefficient under different aspect ratios. Li and Mao [21] calculated the stress intensity factor of the inclined surface crack of the outer wall of the heat exchanger by numerical simulation.

In the research on cracks, it is inevitable to encounter the problem of complete or partial closure of the cracks. For simplicity, more papers have chosen to ignore the surface contact of the cracks. However, the contact closure of the crack face significantly changes the stress–strain distribution at the crack front, thereby affecting the fracture behaviors of the structure. In fact, since the contact area is time-varying, the contact problem is a typical nonlinear problem with boundary conditions. In static fracture mechanics, the contact friction of the crack face has an obvious influence on the stress intensity factor. At present, there have been few studies on mixed mode cracks on the friction surfaces under compressive loading, which have mainly focused on flat plates [22,23,24]. Bowie and Freese [25] proposed a crack closure technique to correct the solution of overlapping crack surfaces, but their method does not consider the sliding between crack surface, and is only suitable for the case of a large friction coefficient. Liu and Tan [26] used the boundary element method to study the effect of the interaction between the friction and crack surface on the stress intensity factor. Hammouda et al. [27,28] used finite element analysis to study the effect of crack surface friction and crack inclination on the stress intensity factor of the central cracked plate under unidirectional compressive load. In addition, Dorogoy and Banks-Sills [29] investigated the effects of loading angle and friction coefficient on the stress intensity factor and the crack length of Brazilian disc cracks under concentrated loading using a finite difference solution.

To the best of the authors’ knowledge, researchers have not focused on the surface crack stress intensity factor of shell structures subjected to compressive loads, however, in engineering practice, fracture damage occurs in shell structures subjected to simultaneous external and axial pressures. In this paper, the stress intensity factors of the shell subjected to simultaneous external and axial pressures were evaluated by using the three-dimensional finite element analysis method, and the effects of the relative depth of the surface cracks, aspect ratio, crack inclination, and friction coefficient on the stress intensity factor of the crack fronts were investigated, which were different from those under internal pressure. The empirical solutions of the mode II and mode III stress intensity factors along the crack front are given using numerical methods, respectively.

## 2. Finite Element Model of Pipe Crack

### 2.1. Meshing of the Pipe with Inclined Crack on the Inner Surface

**Figure 1:**
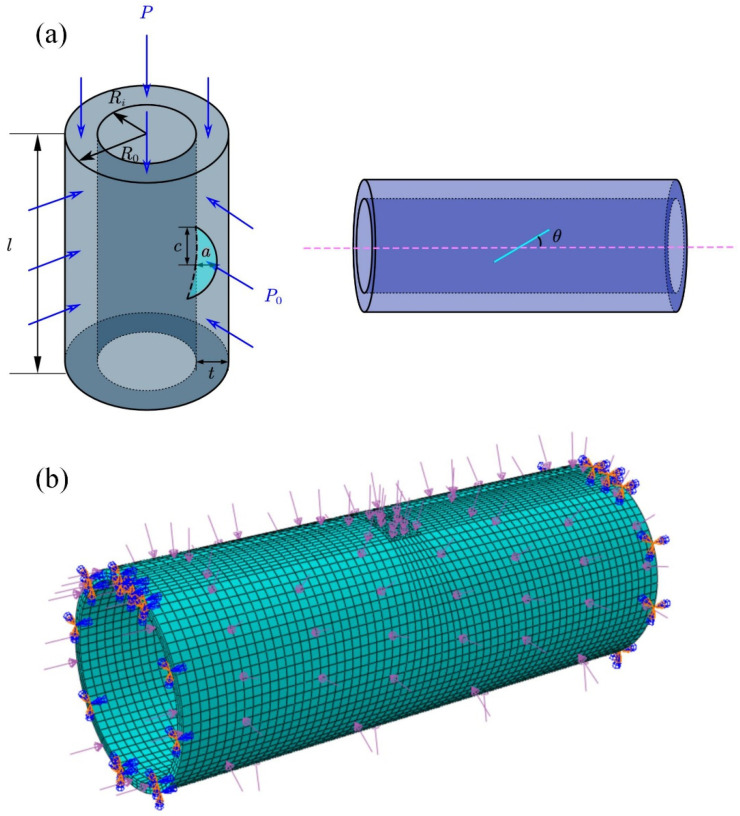
(**a**) Pressure pipe with an inclined crack on the inner surface. (**b**) Loads and boundary conditions.

For the study of mixed mode cracks, the most widely used and accurate method is FEA, which involves the extended finite element method (XFEM) and contour integral. Based on the static loading model in this paper, the fluctuation of results caused by inaccurate meshing can be better avoided by using contour integrals. The commercial code ABAQUS [30] was used for FEA. The material used in this paper was TA2 with a modulus of elasticity of 101,901 MPa and a Poisson’s ratio of 0.348. The pipe size was fixed at *R_i_* = 100 mm, *R*_0_ = 110 mm, *t* = 10 mm. In order to avoid the influence of the boundary effect on the stress intensity factor, the pipe length should be six times that of *R_i_* and 20 times that of the crack half-length c, *l* = 600 mm [31,32]. The crack size is described by dimensionless parameters: the relative crack depth (*a/t*) and the ratio of long and minor axes of semi-elliptical cracks (*a/c*). The crack inclination angle is *θ*. The pipe model is shown in Figure 1a. The external pressure of the pipe *P*_0_ = 100 MPa and the axial pressure *p* = 525 MPa. The boundary conditions were that one end of the pipe was completely fixed and the other end restricted its circumferential constraint, as shown in Figure 1b.

**Figure 2:**
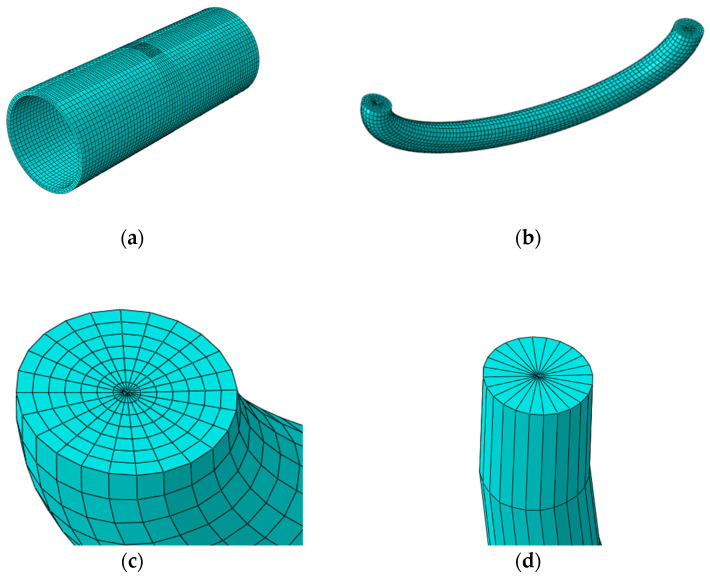
Meshing of the pipe with the inclined internal surface crack:(**a**) global view of the cracked pipe; (**b**) equal divisions at the crack front; (**c**) hexahedron elements around the crack front; (**d**) wedge-shaped elements at the crack front.

The current general semi-elliptical crack meshing method was used by the authors [31,33]. The six-node triangular prism element C3D6 was used to sweep along the path around the crack tip to simulate the stress–strain singularity at the crack front. C3D6 belongs to a fully integrated element with linear interpolation in all directions, the number of nodes is 6, and the number of integration points is 6. The eight-node hexahedral element C3D8R was used in the nearby area. C3D8R belongs to a linear reduced integral element with eight nodes and only one integral point exists in the center of the cell, which is equivalent to a constant stress cell. The reduced integration element can effectively avoid stress discontinuity problems. The meshes at the crack were subdivided, the mesh size was controlled within 1 mm, and the mesh size of the rest zone was controlled within 10 mm to reduce the calculation time. The mesh division is shown in Figure 2.

### 2.2. Penalty Function Method for Contact Problem

Since the pipe is not only subjected to external pressure but also to axial compressive load, contact should be set between the crack surfaces. Generally speaking, the crack surfaces in the contact state have three characteristics:

(1) The contact surfaces do not penetrate or overlap each other;
(2) The contact surfaces are able to transmit normal pressure and tangential friction;
(3) The contact surfaces generally do not transmit normal tension and can be separated freely.

In ABAQUS, the penalty function method, Lagrange multiplier method, and static friction–kinetic friction index decay method can be used to solve the contact problem. In this paper, a penalty function was used to characterize the tangential friction force between the crack surfaces. The penalty function method requires the normal and tangential friction coefficients, which is similar to setting a “spring” between the contact surfaces, and the “spring” works only when the contact surfaces are closed. In this way, the normal contact pressure can be expressed as:

(1) $$P_{n}=\left\{ \begin{array}{l} 0\;\;\;\;\;\;\;\;\;\;\;u_{n}>0\\ k_{n}u_{n}\;\;\;\;\;u_{n}\leq 0 \end{array}\right.$$

**Figure 3:**
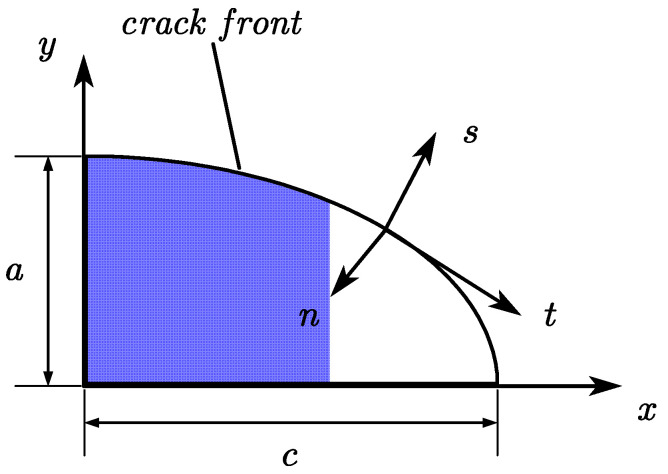
Local coordinate system of the quarter-elliptic crack front.

According to Coulomb’s law of friction, the frictional stress at the contact surface can be written as:

(2) $$\begin{array}{l} \tau_{s}=k_{s}u_{s}-\mu P_{n}\\ \tau_{t}=k_{t}u_{t}-\mu P_{n} \end{array}$$

where *t, n, s* represent the tangential, normal and sub-normal directions, respectively, as shown in Figure 3. The advantage of the penalty function is that it does not increase the degree of freedom of the problem. The disadvantage is that when the friction coefficient is too large, the convergence will become more difficult. The values of the friction coefficient *μ* in this paper were 0, 0.2, and 0.4, respectively.

### 2.3. Mesh Independence Verification

Raju and Newman [2] first proposed a formula to calculate the stress intensity factor of a mode I crack in a pipe, and Chun-Qing Li [19] extended this formula to calculate the mixed-mode stress intensity factor of an inclined crack, as follows:

(3) $$K=\sigma_{0}\sqrt{\pi \frac{a}{Q}}F\left(\frac{a}{t},\frac{a}{c},\frac{t}{R_{i}},\xi ,\theta \right)$$

where *σ*_0_ = *PR/t* is the average hoop stress of the pipe; ***K*** and ***F*** are the stress intensity factor and influence coefficient function under different cracking modes, respectively; *Q* is the shape factor obtained from the second type of elliptic integral; and the empirical formula is given by Shiratori et al. [34].

(4) $$K=\left\{\begin{array}{l} K_{I}\\ K_{II}\\ K_{III} \end{array}\right\}$$

(5) $$F\left(\frac{a}{t},\frac{a}{c},\frac{t}{R_{i}},\xi ,\theta \right)=\left\{\begin{array}{l} F_{I}\\ F_{II}\\ F_{III} \end{array}\right\}$$

(6) $$\begin{array}{ll} Q=1+1.464{\left(\frac{a}{c}\right)}^{1.65} & \frac{a}{c}⩽1\\ Q=\left[1+1.464{\left(\frac{c}{a}\right)}^{1.65}\right]{\left(\frac{a}{c}\right)}^{2} & \frac{a}{c}>1 \end{array}$$

In order to unify the evaluation indicators, it is necessary to normalize the stress intensity factor and the position along the crack front. In this paper, the normalization factor *K*_0_ was defined as [18]:

(7) $$K_{0}=\sigma \sqrt{\pi \cdot a_{0}}\;\;\;\;\;a_{0}=1\;$$

where *σ* = *PR*/2*t* is the axial compressive stress. The position along the crack front can be normalized according to the number of equal meshes. The advantage of this normalization method is that *K_0_* becomes a constant, which can truly reflect the variation trend of *K*.

**Figure 4:**
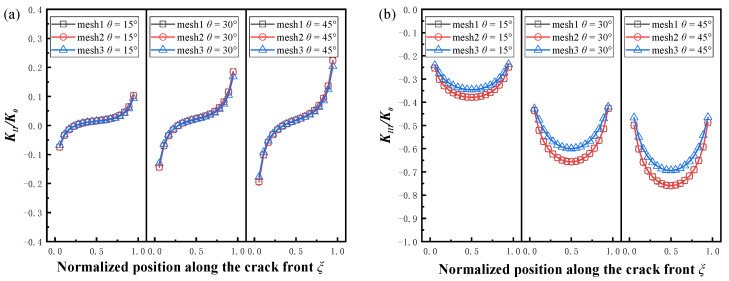
Effect of the mesh density on the normalized SIF at different angles, *a/t* = 0.2, *a/c* = 0.2. (**a**) Normalized *K_II_*, (**b**) normalized *K_III_*.

**Figure 5:**
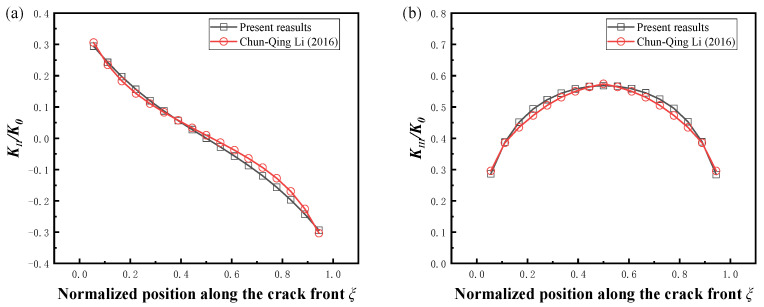
Comparison of the stress intensity factors with mixed modes, *a/t* = 0.2, *a/c* = 0.4, *θ* = 45°. (**a**) Normalized *K_II_*, (**b**) normalized *K_III_* [19].

Figure 4 shows the normalized stress intensity factors at the crack front of three kinds of mesh sizes at different angles. The mesh size is shown in Table 1. It can be seen that the results of the first two mesh sizes were basically the same. A comparison with the normalized *K_II_* and *K_III_* results with Chun-Qing Li [19] after changing the load using mesh2 is given in Figure 5. The average differences were 8.98% and 2.55%, respectively, which demonstrates the accuracy of the results.

### 2.4. Comparison of SIFs between Crack Opening and Crack Closing

**Figure 6:**
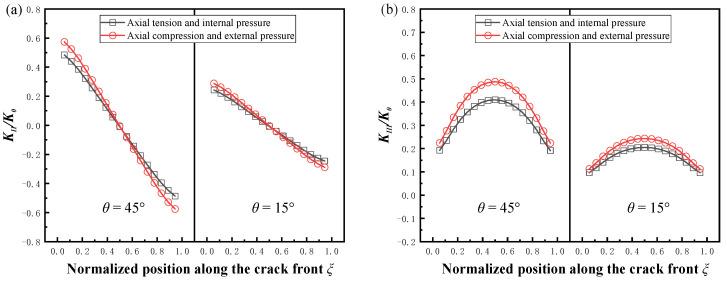
Comparison of SIFs under tension and compression loads with different angles, *μ* = 0*, a/t* = 0.2, *a/c* = 0.8. (**a**) Normalized *K_II_*, (**b**) normalized *K_III_*.

**Figure 7:**
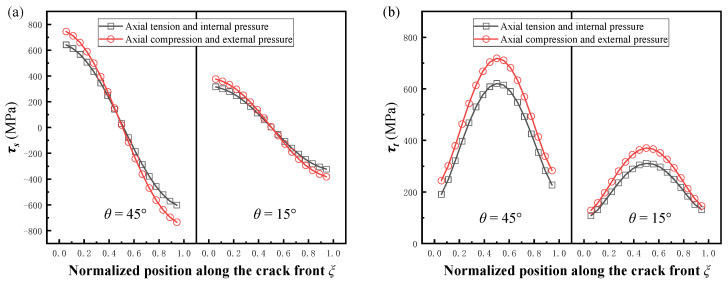
Comparison of shear stress along the crack fronts under tension and compression loads. (**a**) Shear stress along the crack front of mode II. (**b**) Shear stress along the crack front of mode III.

In order to compare the change in the stress intensity factor for crack opening and crack closing, two sets of cracks with different angles were selected in this paper. The contact surface friction coefficient *μ* = 0. The pipe load for crack opening was changed to the internal pressure and axial tension, and the results of the comparison are shown in Figure 6. To facilitate comparison of the differences, the normalized stress intensity factors under external pressure and axial compression were expressed symmetrically about the transverse coordinate. It can be seen that the absolute values of the normalized stress intensity factors at crack closure were obviously higher for both the mode II crack and mode III crack. For this phenomenon, the authors used the stress field calculation method for the plastic zone of the crack tip mentioned by Shlyannikov and Tumanov [35] to convert the three stress states obtained from the finite element calculations to give the sub-normal stresses and tangential stresses at the crack front in these two cases, corresponding to mode II and mode III cracks, respectively, as shown in Figure 7a,b, and it can be seen that *τ_s_* and *τ_t_* at the crack front of the pipe under axial compression and external pressure were higher than those under axial tension and internal pressure, which is the fundamental reason for the difference in SIFs. This difference also proves that it is necessary to study the stress intensity factor of cracks under axial compression load and external pressure.

## 3. Results and Discussion

The stress intensity factor for mode I crack is 0 under the compression load [36,37]. However, due to the crack inclination and external pressure, the *K_II_* and *K_III_* generated by the interaction of tangential and frictional forces at the crack front cannot be neglected. In this paper, the SIFs of pipe surface crack fronts were obtained by FEA under different influencing factors, which included the relative depth (*a/t*), the aspect ratio (*a/c*), the crack inclination angle (*θ*), and the friction coefficient (*μ*). The specific calculation scheme is shown in Table 2. A total of 336 finite element models were calculated in this paper.

### 3.1. Effects of Crack Geometry and Inclination Angle

**Figure 8:**
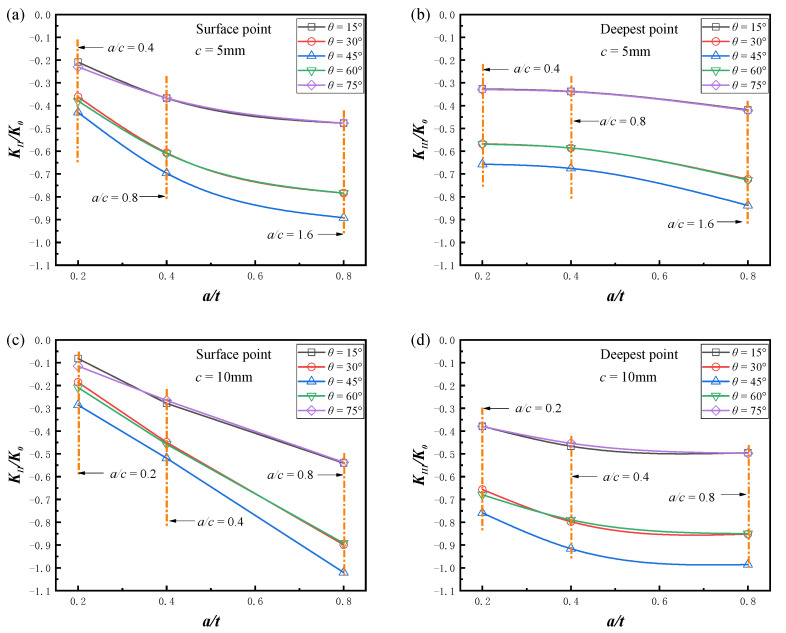
Effect of the relative depth on the normalized SIFs, *μ* = 0. (**a**) Normalized *K_II_* (*c* = 5 mm, *a/c* = 0.4, 0.8, 1.6); (**b**) normalized *K_III_* (*c* = 5 mm, *a/c* = 0.4, 0.8, 1.6); (**c**) normalized *K_II_* (*c* = 10 mm, *a/c* = 0.2, 0.4, 0.8); (**d**) normalized *K_III_* (*c* = 10 mm, *a/c* = 0.2, 0.4, 0.8).

Figure 8 shows the variation in the mode II and mode III SIFs with relative depth. The long axes of the semi-elliptical cracks were fixed as *c* = 5 mm and *c* = 10 mm, respectively, the friction coefficient *μ* was 0. For the convenience of presentation, only the comparison results of the maximum value along the crack front are given, and the rules were consistent for the remaining positions. It can be seen that the absolute value of the mode II and mode III stress intensity factors increased with the deepening of the crack, and the increase in the absolute values was more obvious when the crack size was larger. The surface crack was most dangerous at *θ* = 45° and became safer with the offset of crack inclination to the axial and circumferential directions.

**Figure 9:**
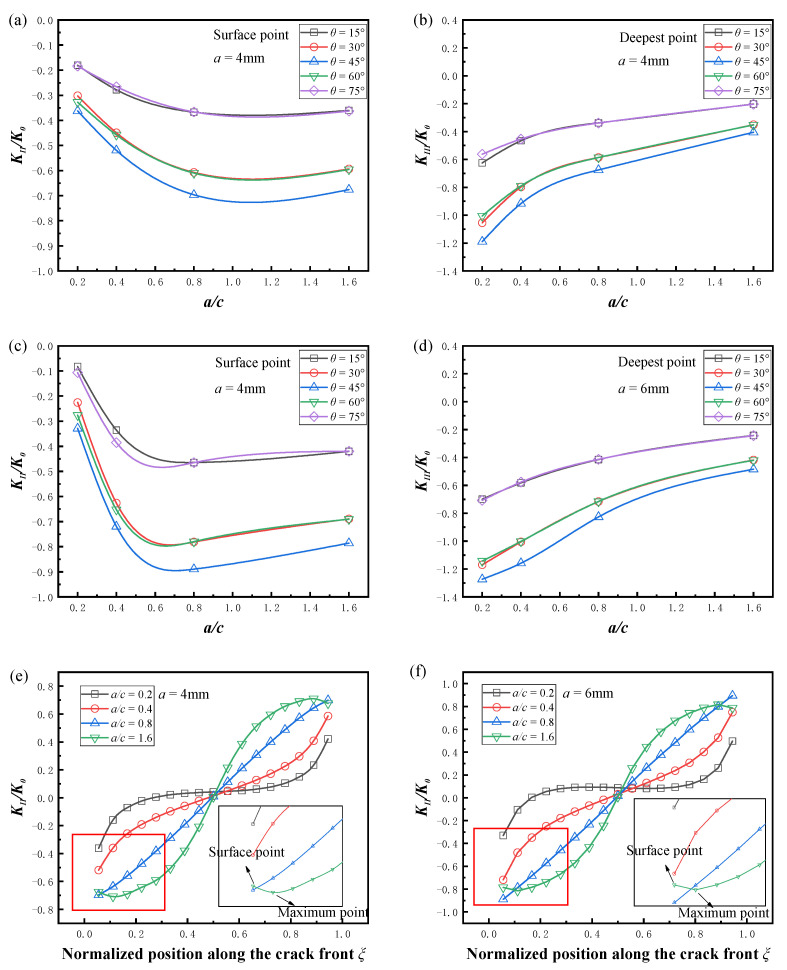
Effect of the aspect ratio on the normalized SIFs (*μ* = 0). (**a**) Normalized *K_II_* (*a* = 4 mm, *a*/*t* = 0.4); (**b**) normalized *K_III_* (*a* = 4 mm, *a/t* = 0.4); (**c**) normalized *K_II_* (*a* = 6 mm, *a*/*t* = 0.6); (**d**) normalized *K_III_* (*a* = 6 mm, *a/t* = 0.6); (**e**) normalized *K_II_* near the crack surface (*a* = 4 mm, *a*/*t* = 0.4); (**f**) normalized *K_II_* near the crack surface (*a* = 6 mm, *a*/*t* = 0.6).

The variation in the mode II and mode III SIFs with the aspect ratio is given in Figure 9, and the minor axis of the semi-elliptical crack was fixed as *a* = 4 mm and *a* = 6 mm, respectively, the friction coefficient *μ* was 0. For the mode II cracks, when *a/c* < 1, the absolute value of the normalized *K_II_* increased with the aspect ratio, and when *a/c* > 1, the absolute value of the normalized *K_II_* decreased with the increase in the aspect ratio. The *K_II_* values of the crack fronts for different aspect ratios at two depths are given in Figure 9e,f. For semi-elliptical cracks with lower aspect ratios, the maximum value of *K_II_* always appeared at the surface point of the crack front. However, for semi-elliptical cracks with higher aspect ratios, the maximum value of *K_II_* appeared near the surface point, which led to a smaller value at the surface point. These variations are consistent with the results of Yang et al. [38] for high aspect ratio cracks. For mode III cracks, the absolute value of *K_III_* decreased monotonically with the increase in aspect ratio, with the most pronounced decrease at *θ* = 45°.

**Figure 10:**
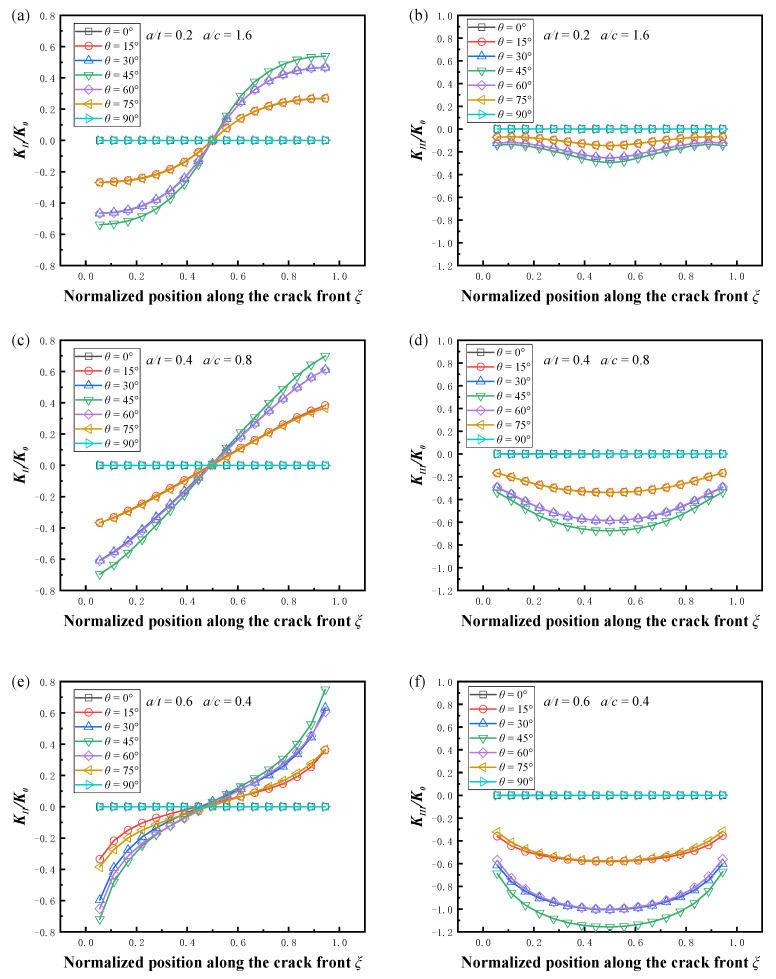
Effect of the inclination angle on the normalized SIFs, *μ* = 0. (**a**) Normalized *K_II_* (*a/t* = 0.2, *a/c* = 1.6); (**b**) normalized *K_III_* (*a/t* = 0.2, *a/c* = 1.6); (**c**) normalized *K_II_* (*a/t* = 0.4, *a/c* = 0.8); (**d**) normalized *K_III_* (*a/t* = 0.4, *a/c* = 0.8); (**e**) normalized *K_II_* (*a/t* = 0.6, *a/c* = 0.4); (**f**) normalized *K_III_* (*a/t* = 0.6, *a/c* = 0.4).

The variation in the mode II and mode III SIFs with the crack inclination angle is given in Figure 10, the friction coefficient *μ* was 0. It can be seen that in all cases, the absolute values of *K_II_* and *K_III_* were symmetrical about the deepest point. The maximum value of the stress intensity factor always appeared at the surface point or the deepest point at *θ* = 45°, and the SIF value was smaller when the crack inclination was closer to the axial and circumferential directions, which was due to the more obvious closure effect under compression load, and the tangential component parallel to the crack surface became smaller. It should be noted that for small-size cracks, *K_II_* and *K_III_* were almost identical when *θ* was symmetric about 45° (*θ* = 30°, 60° and *θ* = 15°, 75°), and the pipe surface cracks at this time could be approximated as flat plate cracks, which has been verified in the study of mixed mode cracks in flat plates and pipes [13,20]. However, for large size pipe surface cracks, the difference caused by the angle was more obvious. The crack surface had a different curvature on the inner wall surface of the pipe at different inclination angles, which made the stress intensity factors no longer consistent for *θ* = 30°, 60° and *θ* = 15°, 75°. As shown in Figure 10e,f, this difference due to curvature was more pronounced in spherical shells [39].

### 3.2. Effect of Friction Coefficient on Contact Surface

**Figure 11:**
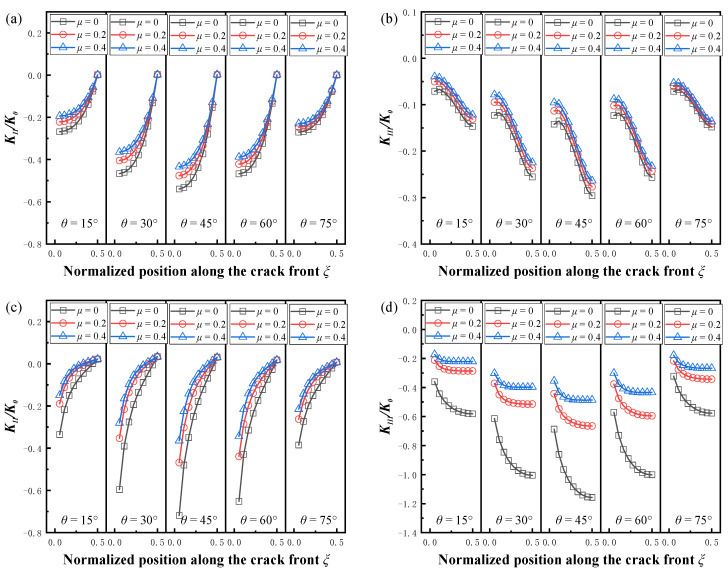
Effect of the coefficient of friction on normalized SIFs. (**a**) Normalized *K_II_* (*a/t* = 0.2, *a/c* = 1.6); (**b**) normalized *K_III_* (*a/t* = 0.2, *a/c* = 1.6); (**c**) normalized *K_II_* (*a/t* = 0.6, *a/c* = 0.4); (**d**) normalized *K_III_* (*a/t* = 0.6, *a/c* = 0.4).

The variation in the mode II and mode III SIFs with the friction coefficient is given in Figure 11. Due to the symmetry of the curves, only the results for the half-length along the crack front are shown in the figure. The absolute values of *K_II_* and *K_III_* decreased with an increasing friction coefficient for different crack sizes and crack inclination angles. At *θ* = 45°, the friction coefficient had the greatest effect on the SIFs, and this effect also increased as the crack size increased. For the mode II cracks, the friction coefficient had the greatest effect on the SIF at the surface point, and this effect decreased as the location of the crack front moved forward, reaching a minimum at the deepest point. In contrast to mode II cracks, the friction coefficient significantly changed the SIF at the deepest point of mode III, but had a dropping effect on the surface point.

## 4. Empirical Solution of SIFs for the Pipe with Inclined Inner Surface Cracks under External Pressure and Axial Compression

Although FEA is one of the most effective methods for calculating SIFs, it is difficult to apply in engineering practice due to the complexity of modeling and time-consuming calculations. In this paper, a new form of empirical solution was proposed by least-squares fitting based on the FEA results to give the influence coefficients at different *a/t* with the normalized crack front position *ξ* as the basis function. A new correction factor for the inclination angle, *f_θ_*, was proposed based on the effect caused by the curvature of the pipe above-mentioned in the effect of the crack inclination angle. The friction coefficient influence coefficient, *f_μ_*, was proposed because the pipe is subjected to external pressure and axial pressure. The influence coefficients are shown in Table 3 and Table 4, and the fitted equations are shown in Equations (8)–(16).

(8) $$K=\sigma \sqrt{\pi \frac{a}{Q}}F\left(\frac{a}{t},\frac{a}{c},\xi ,f_{\theta },f_{\mu }\right)$$

where *σ* is the far-field compressive stress, $K=\left\{\begin{array}{l} K_{II}\\ K_{III} \end{array}\right\}$,$F\left(\frac{a}{t},\frac{a}{c},\xi ,f_{\theta },f_{\mu }\right)=\left\{\begin{array}{l} F_{II}\\ F_{III} \end{array}\right\}$.

Influence coefficient functions for *K_II_*:

(9) $$F_{II}=(H_{1}+H_{2}\xi +H_{3}\xi^{2}+H_{4}\xi^{3})f_{\theta }f_{\mu }$$

(10) $$H_{i(i=1,2,3,4)}=h_{1}+h_{2}(\frac{a}{c})+h_{3}{\left(\frac{a}{c}\right)}^{2}+h_{4}{\left(\frac{a}{c}\right)}^{3}$$

(11) $$f_{\theta }=\left\{1+\frac{ac}{600}\left[\sin\left(\theta -45\right)\right]\right\}\sin2\theta$$

(12) $$f_{\mu }=\left(1+0.575\mu \right){\left(ac\right)}^{-0.505\mu }$$

Influence coefficient functions for *K_III_*:

(13) $$F_{III}=(H_{1}+H_{2}\xi +H_{3}\xi^{2})f_{\theta }f_{\mu }$$

(14) $$H_{i(i=1,2,3)}=h_{1}+h_{2}(\frac{a}{c})+h_{3}{\left(\frac{a}{c}\right)}^{2}+h_{4}{\left(\frac{a}{c}\right)}^{3}$$

(15) $$f_{\theta }=\left\{1+\frac{ac}{1500}\left[\sin\left(45-\theta \right)\right]\right\}\sin2\theta$$

(16) $$f_{\mu }=\left(1+0.281\mu \right){\left(ac\right)}^{-0.511\mu }$$

**Figure 12:**
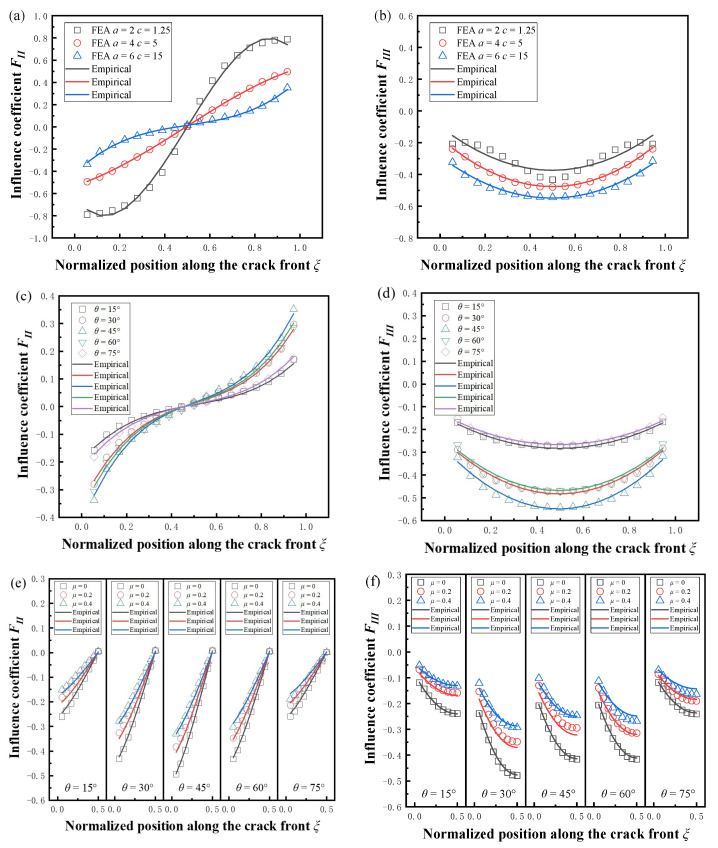
Comparison of SIFs fitting results with (**a**,**b**) different crack sizes (*a/t* = 0.2, 0.4, 0.6, *a/c* = 1.6, 0.8, 0.4, *θ* = 45°, *μ* = 0); (**c**,**d**) different inclination angles (*a/t* = 0.6, *a/c* = 0.4, μ = 0); (**e**,**f**) different friction coefficients (*a/t* = 0.4, *a/c* = 0.8, *μ* = 0, 0.2, 0.4).

The fitting results for *θ* = 45° and *μ* = 0 at the three sizes are given in Figure 12a,b. The results of the fit at different angles for the same crack size are given in Figure 12c,d, where *μ* = 0. The fitting results for the same crack size with different angles and different friction coefficients are given in Figure 12e,f; due to the symmetry of the curves, only the results for the half-length of the crack front are shown in the figure. It can be seen that the fitting results achieved good agreement with the FEA results, which can be applied for the defect assessment.

## 5. Conclusions

In this paper, the SIFs of the inclined crack on the inner surface of the pipe under combined axial pressure load and external pressure were investigated by the finite element method, and the main conclusions are as follows.

(1) By comparing the SIF values along the crack fronts, it can be found that *K_II_* and *K_III_* for the closed surface cracks under external and axial pressure were higher than *K_II_* and *K_III_* for the open surface cracks under internal pressure and axial tension. 

(2) The effects of the relative depth and aspect ratio on the SIFs of the inclined inner crack were analyzed. The results showed that the larger the *a* and *c*, the easier it is for the crack to expand, and when *a/c* >1, *K_IImax_* does not appear at the surface point of the crack, but near the surface point.

(3) The effects of the crack inclination angle on the SIFs of the inclined inner crack were analyzed. The results showed that the SIFs at the crack front were the largest at *θ* = 45°, and their values decreased with an inclination angle toward the axial and circumferential directions. The larger the crack size, the more obvious the asymmetry of the SIFs about *θ* = 45°.

(4) The effects of the friction coefficient on the SIFs of surface crack were analyzed. The larger the friction factor, the smaller the SIFs along the crack front. The friction coefficient had the greatest effect on the surface point of mode II cracks and the deepest point of mode III cracks, and the greatest effect on cracks with *θ* = 45°.

(5) Based on the above results, a new solution for stress intensity factors *K_II_* and *K_III_* were proposed, and the corresponding coefficients for different crack sizes, an angle correction factor *f_θ_*, and a friction correction factor *f_μ_* are given.

## Figures and Tables

**Table 1:** Crack front meshing.

Division Strategy	Mesh1	Mesh2	Mesh3
Wedge element size (mm)	0.05	0.05	0.1
Hexahedral element layers	8	6	3
Equal divisions at the crack front	180	90	45

**Table 2:** Finite element calculation scheme.

Influencing Factors							
*a/t*	0.2	0.4	0.6	0.8			
*a/c*	0.2	0.4	0.8	1.6			
*θ*(°)	0	15	30	45	60	75	90
*μ*	0	0.2	0.4				

**Table 3:** The sub-curve fitting constants in Equation (10) *h_i_*.

	*a/t* = 0.2				*a/t* = 0.4			
Constants	*H*_1_	*H*_2_	*H*_3_	*H*_4_	*H*_1_	*H*_2_	*H*_3_	*H*_4_
*h*_1_	0.161	−0.323	0.159	−0.117	−0.121	1.404	−3.037	1.865
*h*_2_	−2.105	10.376	−18.905	12.616	−0.753	3.320	−7.455	5.953
*h*_3_	1.795	−14.836	34.186	−22.826	0.285	−6.828	21.492	−15.793
*h*_4_	−0.492	4.623	−11.073	7.399	0.020	1.850	−6.686	5.024
	*a/t* = 0.6				*a/t* = 0.8			
Constants	*H*_1_	*H*_2_	*H*_3_	*H*_4_	*H*_1_	*H*_2_	*H*_3_	*H*_4_
*h*_1_	0.338	0.317	−2.580	1.678	0.385	1.134	−5.297	3.484
*h*_2_	−2.987	9.044	−10.429	7.042	−3.309	7.181	−2.875	2.012
*h*_3_	3.129	−14.121	24.981	−16.777	3.648	−13.768	20.741	−13.951
*h*_4_	−0.999	4.641	−8.408	5.654	−1.206	4.955	−8.080	5.433

**Table 4:** The sub-curve fitting constants in Equation (14) *h_i_*.

	*a/t* = 0.2			*a/t* = 0.4		
Constants	*H*_1_	*H*_2_	*H*_3_	*H*_1_	*H*_2_	*H*_3_
*h*_1_	−0.448	−0.582	0.587	−0.497	−1.179	1.171
*h*_2_	0.599	−1.998	2.023	0.727	1.049	−0.907
*h*_3_	−0.322	1.544	−1.596	−0.491	−2.190	1.959
*h*_4_	0.053	−0.313	0.335	0.116	1.001	−0.910
	*a/t* = 0.6			*a/t* = 0.8		
Constants	*H*_1_	*H*_2_	*H*_3_	*H*_1_	*H*_2_	*H*_3_
*h*_1_	−0.451	−0.187	0.210	−0.375	−1.135	1.159
*h*_2_	0.494	−3.521	3.478	0.154	−0.353	0.306
*h*_3_	−0.214	3.819	−3.781	0.200	0.413	−0.373
*h*_4_	0.021	−1.192	1.181	−0.126	−0.058	0.045

## Data Availability

The data that support the findings of this study are available from the corresponding author upon reasonable request.

## Abbreviations

*a*	Depth of a semi-elliptical surface crack
*c*	Half-length of a semi-elliptical surface crack
*F_I_*	Influence coefficient functions for *K_I_*
*F_II_*	Influence coefficient functions for *K_II_*
*F_III_*	Influence coefficient functions for *K_III_*
*f_θ_*	Angle effect correction factor
*f_μ_*	Frictional influence correction factor
*H_1_, H_2_, H_3_, H_4_*	Sub-curve-fitting functions for *F_I_*, *F_II_*, *F_III_*
*h_1_, h_2_, h_3_, h_4_*	Sub-curve fitting constants for *H_1_*, *H_2_*, *H_3_*, *H_4_*
*K_I_*	Stress intensity factors for Modes I crack
*K_II_*	Stress intensity factors for Modes II crack
*K_III_*	Stress intensity factors for Modes III crack
*K_0_*	Normalizing factor for the stress intensity factors
*k_n_*	Main normal stiffness of crack front
*k_s_*	Sub-normal stiffness of crack front
*k_t_*	Tangential stiffness of crack front
*l*	Length of a pipe
*P*	Axial pressure of a pipe
*P_0_*	External pressure of a pipe
*P_n_*	Main normal pressure of crack front
*Q*	Shape factor for elliptical crack
*R_i_,R_0_*	Internal and external radius of a pipe
*t*	Wall thickness of a pipe
*u_n_*	Main normal displacement of crack front
*u_s_*	Sub-normal displacement of crack front
*u_t_*	Tangential displacement of crack front
*θ*	Inclination angle of a surface crack
*ξ*	Normalized position along the crack front
*σ*	Far-field compressive stress
*τ_s_*	Crack front sub-normal shear stress
*τ_t_*	Crack front tangential shear stress
*μ*	Friction coefficient of the cracked surface
